# Preferences for public long-term care insurance among middle-aged and elderly residents: A discrete choice experiment in Hubei Province, China

**DOI:** 10.3389/fpubh.2023.1050407

**Published:** 2023-01-26

**Authors:** He Ma, Erping Jia, Huimin Ma, Yanzhi Pan, Shan Jiang, Juyang Xiong

**Affiliations:** ^1^School of Medicine and Health Management, Tongji Medical College, Huazhong University of Science and Technology, Wuhan, Hubei, China; ^2^School of Population and Public Health, University of British Columbia, Vancouver, BC, Canada

**Keywords:** long-term care insurance, preferences, discrete choice experiment, middle-aged and elderly, China

## Abstract

**Objective:**

It is critical to incorporate residents' preferences into the design of long-term care insurance (LTCI). However, little is known about middle-aged and elderly residents' preferences for personalized need-related attributes of LTCI in China. Through a discrete choice experiment (DCE), we aimed to focus on the direct beneficiaries of LTCI and then elicit their preferences for LTCI under a hypothetical scenario of dysfunction.

**Methods:**

Attributes and levels were defined through a literature review and two rounds of expert consultations (*n* = 8). A D-optimal fractional factorial design was used to generate the DCE questionnaire. Face-to-face interviews with middle-aged and elderly residents were conducted in two cities in Hubei Province, China, between November and December 2020. A mixed logit model was utilized for estimation.

**Results:**

Five attributes were identified and incorporated into the DCE questionnaire. A total of 390 participants completed DCE questionnaires. Care facilities, care content, reimbursement rate, caregivers, and annual premium per person all had a significant impact on residents' preferences. Residents had significantly higher preferences for the LTCI scheme with home and community-based care centers (β = 1.40, *p* < 0.01), multi-level services (β = 0.44, *p* < 0.01), 90% reimbursement rate (β = 0.37, *p* < 0.01), and sufficiently trained caregivers (β = 0.26, *p* < 0.01). Individual characteristics, such as gender, employment, and education level were the factors that drove heterogeneity in preferences for LTCI.

**Conclusion:**

This study provides new evidence on the preferences of middle-aged and elderly residents for personalized need-related public LTCI features. The design of the LTCI scheme in China needs to take these findings into account to maximize the utility for direct beneficiaries of LTCI and enhance their enrollment.

## Introduction

China has witnessed a sharp rise in the need for long-term care (LTC) among older adults. Based on the 2020 national census data, there were ~190 million people aged 65 years old and above in China (1). The proportion of this age group is predicted to double, from 12.5% in 2020 to 27.1% in 2050 (2). The aggravation of population aging, coupled with the growing prevalence of chronic diseases in the elderly, has fuelled a rapid increase in the number of disabled elderly (3). In Hubei Province, the industrial, scientific, and educational center in central China, there were about 0.81 million disabled older adults, accounting for around 8.4% of the population aged 60 years and over (4). In China, the elderly with disabilities primarily seek LTC from their own families and paid professional care facilities (5). The rapid growth of the disabled elderly has triggered a massive need for LTC (6), which imposes a heavy economic burden on both families and society (7, 8). Nevertheless, the elderly with disabilities have limited financial resources to pay for the care (9, 10), therefore, long-term care insurance (LTCI) scheme is explored on need. LTCI is a type of insurance that covers the cost of LTC for the disabled elderly. It comprises both public (primarily provided by government and society) and private (primarily provided by private insurance companies). Given the enormous aging population and their health rights, China's policy is oriented to implementing the social insurance model to develop public LTCI scheme (11). Currently, premiums for LTCI in China are paid by individuals, government subsidies and the health insurance fund. The expenses for the poor who can't afford it are covered by the Chinese government.

China is striving to build an elderly-oriented public LTCI system by 2025 basically (12). In June 2016, the Ministry of Human Resources and Social Security in China issued the “Guidance on Piloting the Long-term Care Insurance.” Jingmen in Hubei Province was one of the first LTCI pilot cities (13). In recent years, the Chinese government has been expanding the LTCI pilot (14). Under this trend, Wuhan, the capital city of Hubei Province, intends to accelerate the establishment of the LTCI system (15, 16). As of 2021, China had been piloting the public LTCI in forty-nine cities, covering 140 million people (17), which had been proven successfully in many aspects (18, 19), but also been found several challenges. In pilot cities, care content such as psychological comfort has been absent from the LTC service, and the fundamental role of home and community-based care centers has not been adequately played (20). A survey study finds that the average bed occupancy rate of care facilities hit 64% with an almost 40% vacancy rate (21). Some studies point out that the criterion for determining the level of treatment payment remains to be strengthened (20, 22). In addition, due to the shortage of trained caregivers, the LTC needs of almost 40 million disabled elderly cannot be met effectively (6).

To address these issues, it is crucial for the LTCI scheme to identify and incorporate residents' preferences into its initial design (23–26). Nevertheless, only limited preference information can be drawn from non-experimental methods (27, 28). Stated preference elicitation methods such as discrete choice experiment (DCE) can be a useful tool for preference elicitation (29, 30). It has been widely utilized in the field of health economics and policy research (31, 32). Studies in Thailand and other countries have applied this method to exploring individual preferences for attributes of LTCI and other health insurances (33–37). In the field of DCE research on LTCI in China, He et al. have focused on middle-aged adults' needs for the product-related attribute of private LTCI (38). Wang et al. have explored residents' preferences for the financial affordability-related attribute of public LTCI (11). To the best of our knowledge, there is no published study exploring middle-aged and elderly preferences for personalized need-related attributes of public LTCI in China.

To provide new insights into studying residents' preferences for LTCI, our study is to elicit and quantify middle-aged and elderly residents' preferences for personalized need-related attributes of LTCI using a DCE. We also assess the relative importance (RI) that middle-aged and elderly residents place on different attributes. The results of this study would provide evidences for designing and enhancing the LTCI scheme for the middle-aged and elderly residents.

## Materials and methods

DCE has been used to elicit preferences for attributes of a specific product and could be a useful tool for revealing the critical aspects of LTCI choice decisions (39). A DCE design consists of the following stages (40): (1) determining the key attributes and their levels, (2) selecting the experimental design method and constructing the choice sets, (3) developing questionnaires, pre-survey, collecting data, (4) data entry, and (5) data analysis and interpretation.

### Establishing the attributes and levels of the DCE

To select the attributes and levels for this study, a literature review and two rounds of expert consultations were conducted (shown in Supplementary material). First, an initial list of attributes was created based on existing health policy documents and literature reviews (12–14, 27, 28, 33, 41–43). Second, eight experts (three medical security experts, four health policy research experts, and one health economics expert) were invited to evaluate the list. On the basis of two rounds of experts' revision opinions and their ratings of the importance and feasibility of attributes, we refined each attribute and its levels. Finally, five attributes were used to define the key personalized need-related features of the hypothetical LTCI schemes. According to a recommendation from an ISPOR report (39), each attribute in this study was set to three levels. Table 1 displays the selected attributes and levels. The detailed explanations were as follows.

**Table 1:** Attributes and levels.

**Attributes**	**Levels**
Care content	Daily life care^a^
	Daily life care and rehabilitation
	Daily life care, rehabilitation and emotional support
Care facilities	Rehabilitation hospitals^a^
	Nursing homes
	Home and community-based care centers
Caregivers	Basically trained caregivers^a^
	Moderately trained caregivers
	Sufficiently trained caregivers
Annual premium per person	30CNY^a^
	60CNY
	90CNY
Reimbursement rate	50%^a^
	70%
	90%

^a^Reference level for each attribute; CNY, Chinese Yuan; the average annual exchange rate between USD and CNY in 2020 was 1CNY = 0.155 USD (44) (International Monetary Fund data).

The first attribute is care content, which refers to the service types of LTC. As a previous study has indicated, rehabilitation and emotional support have been cited as important service items for elderly people (45). However, in pilot cities, daily life care is provided mainly while personalized services (e.g., emotional support) have not got much attention (46, 47). Considering the personalized needs of the disabled elderly, three types of services were chosen and combined to produce three gradually extended services. In this attribute, daily life care includes living services (e.g., dressing and washing) and instrumental services (e.g., cleaning and purchasing). Rehabilitation refers to health consultation and management, medication guidance, etc. provided by trained nursing staff. Emotional support consists of chatting, escorting, counseling etc.

The second attribute is care facilities, which refers to LTC institutions settings. We set three levels for this attribute. The first level represents medical institutions that primarily provide rehabilitative care. The second level shows welfare institutions which dedicate to providing centralized accommodation and nursing care. The third level refers to home and community centers that can provide in-home care or community-based day care for insured residents.

The third attribute is caregivers, which indicates long-term nursing staffs with different professional skill levels. The low care quality and unskilled health workers were the negative predictors for the elder to enrollment in nursing homes (29, 48). According to the national vocational skill standards (49), we defined three attribute levels based on the degree to which caregivers got skills training.

The next two attributes are reimbursement rate and the annual premium per person. In DCE studies on insurance preferences of residents, attributes on the cost were always included (35, 50–53). The inclusion of the annual premium per person attribute allows for the estimation of residents' willingness to pay for improvements in other attributes. According to Jingmen City's financing standard (43) and expert consultations, we set three levels for the annual premium per person. In this study, the reimbursement rate represents the ratio that the LTCI can cover the nursing expenses for elderly persons with disabilities. Three levels were defined for this attribute, with 70% as the center level (12) and 20% above and below. We use percentage changes rather than levels because the reimbursement ratio varies substantially in the pilot cities.

### Experimental design and development of the questionnaire

The combination of these attributes and levels (five attributes with three levels) resulted in 243 hypothetical scenarios (3^5^ = 243). To maximize the efficiency and precision of the design, we generated a D-optimal fractional factorial design using the %ChoiceEff macro in SAS 9.4 software (54). 18 choice sets were developed and randomized to 2 versions of the survey, each with two alternatives, to minimize respondent burden. Within each version, the seventh choice set was duplicated to examine the internal consistency and rationality. An example choice set is shown in Table 2. Since the whole population of Jingmen City participated in the long-term care insurance, we simulated this real situation in Wuhan City. Therefore, no opt-out option was included in our study.

**Table 2:** Example of the choice set.

**Attributes**	**LTCI**	**LTCI**
Care content	Daily life care and rehabilitation	Daily life care, rehabilitation, and emotional support
Care facilities	Home and community-based care centers	Rehabilitation hospitals
Caregivers	Moderately trained caregivers	Basically trained caregivers
Annual premium per person	60 CNY	30 CNY
Reimbursement rate	70%	90%
Which one do you prefer?	○	○

In addition, the questionnaire contained questions about the demographic characteristics (e.g., gender, age, education level, and living condition) and attitudes toward the risk of dysfunction. To check the understandability of the DCE questionnaire, we also conducted a pilot test comprising 20 residents selected from a community in Wuhan and revised the questionnaire based on the feedback from respondents. The pilot test suggested that the process of administering the questionnaire took about 20–25 min on average.

### DCE implementation and data collection

Following the sample size calculation methods proposed by Johnson and Orme (55), 84 respondents would provide adequate power to detect the main effects. Considering that a large sample size would facilitate heterogeneity analysis, we targeted a sample of 400 residents aged 45 and over in Hubei Province, the typical provincial capital in central China. Jingmen City is the first batch of LTCI pilot cities in the country, and Wuhan City, the capital of Hubei Province, has the potential to be the province's next pilot city (56, 57). Therefore, we selected these two representative cities as experimental implementation sites. Then, the survey was conducted in Qiaokou District of Wuhan City and Dongbao District of Jingmen City, according to the distance of field research and the convenience of data acquisition. Next, two communities were chosen randomly from each district. Finally, we selected one hundred residents aged 45 years and older in each community.

Data were collected from November to December 2020 and 390 respondents completed the questionnaire. The local health bureau assigned study coordinators specialized in health insurance management from Wuhan City and Jingmen City to help us recruit respondents. The study coordinators screened the residential databases to find eligible participants. They contacted the eligible participants in advance by phone calls or WeChat groups to check their availability to complete the questionnaire. Before data collection, the interviewers were given intensive and uniform training and were able to get respondents across the meaning of the LTCI schemes. Residents were invited into communities to complete the questionnaire anonymously through in-person interviews. They were reminded of making choices under a hypothetical dysfunction scenario (a situation when people are old, with poor physical health, unable to take care of themselves, and have an urgent need for long-term care). Each resident was given a gift valued about 16 CNY ($2.48) for participation. Participants were informed that participation was voluntary and that completing the questionnaire indicated informed consent.

### Data analysis

We estimated the relative priority respondents placed on each of the attributes using mixed logit model which is a popular method for studying the DCE (58). Dummy coding was applied to four attributes other than annual premium per person, which was defined as numeric continuous variable in the mixed logit model. In our model, expected utility U that resident *i* obtained from an LTCI alternative *j* in the choice set *t* was given by:


$$\begin{array}{l} U_{ijt}=V_{ijt}\left(X_{ijt},\beta \right)+\varepsilon_{ijt}\;;\;i=1,\ldots ,390;\;j=1,2;\;t=1,\ldots ,9\; \end{array}$$

Where *V*_*ijt*_ indicates the fixed term of *U*_*ijt*_, ε_*ijt*_ is the error term, *X*_*ijt*_ is a vector of variables representing the attributes of alternative *j* and β is a vector of coefficients. The mean value and standard deviation appeared in the model estimation results. In this study, we estimated the main effects of the mixed logit model in the first stage, and then we estimated models with interaction terms to assess potential differences in preferences across groups with different sociodemographic characteristics. We calculated the RI of each attribute by dividing the difference in utility between the lowest and highest level of that attribute by the sum of the difference for all attributes (59, 60). We also calculated the willingness to pay (WTP) for the attribute levels using the estimated coefficients divided by the coefficient of the annual premium per person. In addition, we performed subgroup analysis to examine whether residents' preferences differed systematically based on sampling areas and dysfunctional risk attitudes. All analyses were performed using Stata statistical software (V.12 SE, StataCorp). Statistical significance was set at α = 0.05.

## Results

### Characteristics of respondents

Since no consensus in the literature that failing the internal consistency test indicates irrationality (61, 62), we also analyzed data that failed. A total of 390 residents participated in the survey. Table 3 presents some of their characteristics, compared to the general population. The age structure varies between the sample and the general population. Over half or more than the respondents were aged 65 years and over. The majority of respondents were female (72.13%). More than 80% of respondents lived in rural areas. The majority of respondents (almost 85%) are married. Over 90% of respondents had at least one child. In terms of employment, the retirees (53.08%) accounted for the highest proportion. The vast majority of the population (99.23%) was covered by medical insurance schemes. Half of the respondents had an education level with middle school and below. Less than 10% of the population lived alone. Most respondents (92.82%) lived with spouses or children. The average monthly household income was 4,000 CNY (620.8$).

**Table 3:** Respondents characteristics (*n* = 390).

**Characteristics**	**Sampling residents *n* (%)**	**The general population in Hubei %**
**Area**		
Wuhan City	201 (51.54)	N/A
Jingmen City	189 (48.46)	N/A
**Region**		
Urban area	316 (81.03)	62.89
Suburban area	74 (18.97)	37.11
**Gender**		
Male	108 (27.69)	34.61
Female	282 (72.31)	65.39
**Age**		
45–65	187 (47.95)	68.37
≥65	203 (52.05)	31.63
**Marital status**		
Married	331 (84.87)	72.72
Unmarried (e.g., never married, divorced, and widowed)	59 (15.13)	27.28
**Children**		
0	10 (2.56)	N/A
1	205 (52.56)	N/A
≥2	175 (44.87)	N/A
**Living condition**		
Live alone	28 (7.18)	10.71
Live with others (e.g., parents, spouse, children, and siblings)	362 (92.82)	89.29
**Education level**		
Middle school and below	197 (50.51)	60.84
High and secondary school	103 (26.41)	20.64
Junior college	52 (13.33)	9.72
Bachelor or above	38 (9.74)	8.80
**Employment**		
Employed/working	107 (27.44)	N/A
Retiree/pensioner	207 (53.08)	N/A
Not working	76 (19.49)	N/A
Average monthly household income	4,000 (2,500, 6,000)	N/A
**Insurance**		
Yes	387 (99.23)	N/A
No	3 (0.77)	N/A

The characteristics information of the general population in Hubei was retrieved from China Statistical Yearbook 2020 (63).

### Discrete choice experiments model estimation

Twenty five respondents did not pass the consistency test. To verify the robustness of the results, this study excluded the 25 respondents and then analyzed the total samples. The results are presented in Supplementary Table 1. There was no change in the statistical significance (α = 0.05) of the estimated model parameters for each attribute before and after exclusion. Therefore, we included all 390 respondents in the analysis.

Table 4 presents the results of the mixed logit model. Respondents highly valued home and community-based care centers (β = 1.40, *P* < 0.01), followed by multi-level nursing services that include daily life care, emotional support, and rehabilitation (β = 0.44, *P* < 0.01). The reimbursement ratio that covered 90% of nursing expenditures was also a significant and positive predictor (β = 0.37, *P* < 0.01), while premium level per person was a significant and negative predictor (β = −0.01, *P* < 0.01). And nursing homes was a significant and negative predictor (β = −0.51, *P* < 0.01). Four variables, namely care content, care facilities, caregivers, and reimbursement rate, were found to have unobservable preference heterogeneity as indicated by the estimated SD of the coefficients.

**Table 4:** Mixed logit main-effect model estimates and willingness to pay (*n* = 390).

**Attributes**	**β**	**SD**	**WTP**
**Care content (ref: daily life care)**			
Daily life care and rehabilitation	0.06	0.14	6.44 (−5.96, 18.84)
Daily life care, rehabilitation and emotional support	0.44^*^	0.36^*^	48.61 (30.51, 66.71)
**Care facilities (ref: rehabilitation hospitals)**			
Nursing homes	−0.51^*^	0.96^*^	−56.18 (−78.75, −33.61)
Home and community-based care centers	1.40^*^	1.59^*^	154.98 (100.38, 209.58)
**Caregivers (ref: basically trained caregiver)**			
Moderately trained caregiver	−0.02	0.05	−1.80 (−13.64, 10.03)
Sufficiently trained caregiver	0.26^*^	0.29^*^	28.99 (14.12, 43.87)
**Reimbursement rate (ref: 50%)**			
70%	0.08	0.03	9.11 (−1.21, 19.44)
90%	0.37^*^	0.63^*^	41.06 (21.69, 60.43)
Annual premium per person (CNY)	−0.01^*^		
Log-likelihood	−1,767.58		
Participants	390		
Observations	7,020		

β, the average preferences of the study population; SD, standard deviation; WTP, willingness to pay; Ref, reference level; ^*^p < 0.01.

Based on the analysis of stated preference, our study found that respondents would be willing to trade 154.98 CNY ($24.05) for community-home care centers, followed by 48.61 CNY ($8.54) for multi-level nursing care that includes daily life care, emotional support, and rehabilitation. The value for reimbursement ratio of 90% was 41.06 CNY ($6.37) and the value for sufficiently trained caregivers was 28.99 CNY ($4.50). Residents had negative WTP for selecting nursing homes as care facilities.

### Preference heterogeneity

Table 5 shows the results of the preference heterogeneity analysis. In the hypothetical dysfunction scenarios, some demographic attributes played significant roles in decision-making: gender, employment, education level, and average monthly household income. The negative coefficient of the interaction between employment and nursing homes (β = −0.42, *P* < 0.05) indicated that respondents who retired or off working attached less utility to nursing homes. Female respondents attached more utility to home and community-based care centers compared with male residents (β = 0.66, *P* < 0.01). Respondents with a higher education level valued the annual premium per person more (β = 0.01, *P* < 0.01). Higher monthly household income respondents placed a higher value on the annual premium per person (β = 0.01, *P* < 0.01).

**Table 5:** Results of the preference heterogeneity analysis.

**Attributes**	**β**	**95%CI**
**Care content (ref: daily life care)**		
Daily life care and rehabilitation	−0.26	(−0.78, 0.26)
Daily life care, rehabilitation and emotional support	0.62^**^	(0.14, 1.10)
**Care facilities (ref: rehabilitation hospitals)**		
Nursing homes	0.14	(−0.49, 0.76)
Home and community-based care centers	0.52	(−0.39, 1.43)
**Caregivers (ref: basically trained caregivers)**		
Moderately trained caregivers	−0.11	(−0.61, 0.39)
Sufficiently trained caregivers	0.12	(−0.39, 0.63)
**Reimbursement rate (ref: 50%)**		
70%	0.24	(−0.19, 0.68)
90%	0.27	(−0.31, 0.86)
Annual premium per person (CNY)	−0.03^**^	(−0.05, −0.02)
**Interaction terms**		
Gender ^*^ home and community-based care centers	0.66^**^	(0.21, 1.10)
Education level ^*^ annual premium per person	0.01^**^	(0.004, 0.012)
Employment ^*^ nursing homes	−0.42^*^	(−0.81, −0.03)
Employment ^*^ home and community-based care centers	0.81^**^	(0.24, 1.38)
Average monthly household income ^*^ annual premium per person	0.01^**^	(0.003, 0.020)
Log-likelihood	−1,720.70	
Participants	390	
Observations	7,020	

β, the average preferences of the study population; CI, confidence interval; Ref, reference level; ^*^p < 0.05; ^**^p < 0.01.

### Relative importance of attributes

**Figure 1:**
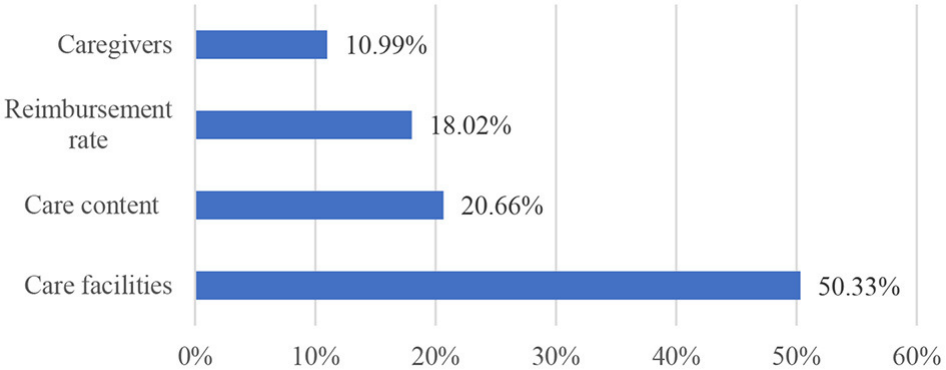
Relative importance of the attributes.

Figure 1 illustrates the RI of the attributes. The care facility was the most preferred LTCI attribute (50.33%) among respondents, followed by care content (20.66%), reimbursement rate (18.02%), and caregivers (10.99%).

### Subgroup analysis

Supplementary Table 2 presents the results from the subgroup analysis by area (Wuhan City vs. Jingmen City). While the two groups had similar results, there were some differences worth noting. For example, although nursing homes remained a significant negative predictor, the coefficient for residents in Jingmen City was −0.21 (*P* < 0.01), not as important as for respondents in Wuhan City (β = −0.86, *P* < 0.01). The expected gains in health outcomes from the sufficiently trained caregivers seemed to be more important for respondents in Wuhan City (β = 0.36, *P* < 0.01) when compared to those in Jingmen City (β = 0.16, *P* < 0.05). As for the WTP for care content, respondents from Wuhan City would be willing to pay 54.45 CNY ($8.45) more for multi-level care services than those from Jingmen City respectively.

Supplementary Table 3 shows the subgroup analyses by attitudes to the risk of dysfunction. The coefficient of annual premium per person was negative significant for respondents with neutral/pessimistic attitudes to anticipatory dysfunction, but not for those with optimistic attitudes. The nursing home also had negative significant coefficients for respondents with optimistic/neutral attitudes toward anticipatory dysfunction, but not for those with pessimistic attitudes. The coefficient of “daily life care and rehabilitation” was positively significant for respondents with optimistic attitudes, while those with neutral/pessimistic attitudes were more inclined to choose service packages that include emotional services. Respondents with optimistic attitudes would be willing to pay 386.41 CNY ($57.18) and 476.75 CNY ($74.00) more for community and home-based care centers than those with neutral/pessimistic attitudes respectively.

## Discussion

To our knowledge, this is one of the first studies to elicit and quantify LTCI preferences of residents aged 45 years and over in China. This study focused on the direct beneficiaries of LTCI and finally identified attributes that reflected their personalized needs. Some of our results are complementary to prior research in which identified attributes more reflected individual' expected financial reimbursement from insurance (11). By eliciting preferences for a hypothetical LTCI scheme, this study has also revealed interesting findings that can inform China's LTCI system reforms within the multi-level social security framework. As the results indicated, all the LTCI features (attributes) included in our DCE were identified as important by respondents. The most valued features were care facilities, followed by care content, reimbursement rate, and caregivers. The above results held basically following subgroup analysis testing. Individual characteristics such as gender, employment, education level, and attitudes to the risk of dysfunction also played crucial roles in preference decisions.

One interesting finding was that the care facility was ranked as the most valued LTCI feature by residents. Residents favored community and home-based care centers (β = 1.40, *p* < 0.01). This mirrors the Chinese culture of filial piety and the cheaper cost of home care services (64, 65). The result supported evidence from an empirical study in Qingdao city (66) and a DCE study in northeast China (11), which indicated that people preferred to access home and community-based care services. However, due to the low level of treatment payments and the lack of financial subsidies for home caregivers, home care function is weakened (21, 67). Therefore, to cater to the primary needs of residents, LTCI schemes should be designed to encourage the development of home and community-based care centers.

Care content was the second most valued LTCI feature among residents. Residents preferred multi-level care (daily life care, rehabilitation, and emotional support) (β = 0.44, *p* < 0.01). This result was aligned with findings from previous DCE studies in Thailand, which also reported coverage of health services as a key factor influencing consumers' health insurance choices (33). A prior study suggested that LTCI in China started from covering simple benefit packages and should consider the potential for later expansion (11). Our findings show that LTCI schemes covered rehabilitation and emotional care were highly valued, in line with prior evidence (45) and further indicating possible directions for pilot expansion of LTCI packages. The shortage of health workforce is a worldwide problem (68). With rising care recipient disability and need for care, the caregiver's role becomes more labor-intensive, time-consuming, and complex (69). In China, there is a vast gap between the supply and demand of caregivers (6), which may affect residents' preference for LTCI. Our findings indicate that it is of great necessity for the Chinese government to train and support caregivers (70) to enable them to deliver adequate and high quality LTC services.

Reimbursement rate was the third most important LTCI feature for residents, ranking behind care content. This finding suggested that residents were likely to pay less attention to cost attributes, such as reimbursement, once they were guaranteed adequate coverage in terms of care services content. This is consistent with prior DCE results in Ethiopia (25), suggesting that respondents were willing to pay more for higher-benefit insurance plans. Moreover, we observed that residents favored a higher rate of reimbursement. This was similar to the result of a previous DCE research where respondents prefer higher-coverage LTCI (11). Furthermore, the result also showed that lower reimbursement negatively affected preferences for LTCI (11). During data collecting, some residents could not understand why they would still have to pay a portion of the nursing costs once insured. These findings suggest that, while deliberatively increasing reimbursement rates, it is essential to help residents raise health insurance awareness.

Another interesting finding of our study was that moderately trained caregivers had a significant and negative coefficient (β = −0.02, *p* < 0.01), while the other two categories of caregivers had significant and positive coefficients. This result implies that moderately trained caregivers in LTCI service delivery may confront embarrassing conditions. On one hand, family caregivers, though less trained, are geographically and emotionally closer to care recipients (71). On the other hand, those with sufficient training possess more comprehensive skills, such as being suited to provide proportionate amounts of emotional and instrumental support (72), and are more equipped to deliver the long-term care older consumers prefer and deserve (73). In general, moderately trained caregivers have no outstanding advantages compared to other caregivers. Therefore, there is a need for the government to invest additional resources in training insufficiently trained caregivers to develop caregivers capable of delivering high-quality and comprehensive LTC services.

The WTP calculation results revealed that residents were more willing to pay for home and community-based care centers than other care facilities, and were willing to avoid a nursing home admission. This result may be due to the high availability of home healthcare services in China lowered the willingness to accept elder care institutions (74). This may also be an indication that nursing home are not fulfilling their functional role, resulting in residents deriving less utility from them. Regarding the care content, residents preferred multi-level services rather than single services, with WTP for multi-level services being almost eight times higher than WTP for services lacking emotional support. This result was similar to a prior study finding that respondents were prepared to pay eight times more for services including social time than those including the same amount of cleaning time (75). In terms of WTP for reimbursement rates, residents preferred higher reimbursement rates. The reason may be that the increase in medical insurance reimbursement rates boosted the elderly's WTP for home care services (76).

Apart from these, we found that preference heterogeneity existed across residents with different individual characteristics. The retired or unemployed, female residents were more inclined to choose home and community-based care centers. The results may be related to the Chinese culture that female residents are more inclined to get emotional support from relatives to meet their affection needs (77, 78). Our study also found that residents with higher education level attached more utility to a higher level of premium. The results are not surprising as education level influenced the structure of knowledge and ideology. In addition, those with more positive attitudes toward the risk of dysfunction had higher (or lower) preference for LTCI features. This finding highlights the necessity to cultivate awareness of health risk prevention. Taken together, these results suggest that, to maximize residents' utility and enhance enrollment in LTCI schemes, it is indispensable to consider the differences in preferences of the elderly population with different individual characteristics.

Some limitations should be noted in this study. First, these five key attributes in the design may not fully reflect residents' decisions in the real world. Decision-making on LTCI is complex. Choosing an optimal LTCI scheme requires evaluating all programs' benefits and costs as well as carefully studying other key issues. Second, in many countries (e.g., Northern Europe), LTCI is primarily funded by tax revenues. In China, LTC's funding comes from multiple channels, that is, it has diverse funding responsibility entities. Therefore, the generalizability of this study is limited to those countries where LTCI is financed by individual out-of-pocket, social insurance, and other responsible subjects. Third, there are some limitations in the selection of respondents. The establishment of the LTCI system will refer to the way of raising medical insurance funds, and a certain proportion of funds of LTCI may be deducted from the wages of workers. That means workers under the age of 45, or urban and rural residents would also have to pay for LTCI but were excluded from the study. In addition, while the research was supported by the local health bureau, the contact databases which were available for us to invite participants only represent specific population groups, especially the older age groups, registered with family doctors. Last, potential bias may exist in our study. The high accessibility to the women and the elderly in China may lead to differences in the gender and age structure of the sample and the overall.

## Conclusion

In this study, we attempted to examine preferences for public LTCI by residents aged 45 years and over in China. Our results highlighted the RI of LTCI features and WTP for each attribute level. LTCI features regarding the care facilities, care content, and reimbursement rate, were valued highly by middle-aged and elderly residents. As well, middle-aged and elderly residents placed different RI on these factors among different sociodemographic characteristics. Developing a multi-level long-term care service system, delivering comprehensive and high-quality LTCI services, cultivating health risk prevention awareness, and heightening individual awareness of health insurance would elicit potential LTCI seeking and increase utility among middle-aged and elderly residents. This study provides evidences for policy making on designing and enhancing the LTCI scheme for the middle-aged and elderly residents.

## Data availability statement

The original contributions presented in the study are included in the article/Supplementary material, further inquiries can be directed to the corresponding author.

## Ethics statement

Ethical review and approval was not required for the study on human participants in accordance with the local legislation and institutional requirements. Written informed consent for participation was not required for this study in accordance with the national legislation and the institutional requirements. Participants were informed that participation was voluntary and that completing the questionnaire indicated informed consent.

## Author contributions

HeM and EJ contributed to study design, statistical analysis, and initial drafting and revision. HuM and YP contributed to investigation and validation. JX and SJ reviewed the manuscript. JX guided the entire process. All authors contributed to the article and approved the submitted version.
