# Supplementary material for: Preferences for public long-term care insurance among middle-aged and elderly residents: A discrete choice experiment in Hubei Province, China
Source: Front Public Health. 2023 Jan 26;11:1050407. doi: 10.3389/fpubh.2023.1050407 (PMC9909219; doi:10.3389/fpubh.2023.1050407)
Supplement: Supplementary file 1 [file Data_Sheet_1.docx]

Supplementary Material

# Supplementary Tables

**SUPPLEMENTARY TABLE 1⎪** Mixed logit main-effect model estimates and willingness to pay (n=365).

| **Attributes** | **β** | **SD** | **WTP** |
| --- | --- | --- | --- |
| **Care content (ref: daily life care)** |  |  |  |
| Daily life care, rehabilitation | 0.06 | 0.24 | 6.45(-6.52,19.41) |
| Daily life care, rehabilitation and emotional support | 0.44^**^ | 0.40^**^ | 48.97(30.10,67.86) |
| **Care facilities (ref: rehabilitation hospitals)** | | | |
| Nursing homes | -0.51^**^ | 0.97^**^ | -55.83(-79.16, -32.50) |
| Home and community-based care centers | 1.38^**^ | 1.56^**^ | 152.18(96.86,207.49) |
| **Caregivers (ref: basically trained caregivers)** | | | |
| Moderately trained caregivers | -0.03 | 0.09 | -3.50(-15.88,8.88) |
| Sufficiently trained caregivers | 0.25^**^ | -0.26^*^ | 27.06(12.16,41.97) |
| **Reimbursement rate (ref: 50%)** |  |  |  |
| 70% | 0.09 | 0.07 | 10.36(-0.53,21.26) |
| 90% | 0.36^**^ | 0.62^**^ | 40.12(20.41,59.84) |
| **Annual premium per person(CNY)** | -0.01^**^ |  |  |
| **Log-likelihood** | -1659.19 | | |
| **Participants** | 365.00 | | |
| **Observations** | 6570.00 | | |

*β, the average preferences of the study population; SD, standard deviation; WTP, willingness to pay; Ref, reference level; *p＜0.05; **p＜0.01.*

**SUPPLEMENTARY TABLE 2⎪** Subgroup analysis by areas.

| **Attributes** | **Wuhan City** | | **Jingmen City** | |
| --- | --- | --- | --- | --- |
|  | **β** | **WTP** | **β** | **WTP** |
| **Care content (ref: daily life care)** | |  |  |  |
| Daily life care, rehabilitation | 0.09 | 17.46(-17.20,52.11) | 0.04 | 3.23(-9.39,15.84) |
| Daily life care, rehabilitation and emotional support | 0.49^**^ | 91.75(15.25,168.26) | 0.40^**^ | 32.30(18.27,46.33) |
| **Care facilities (ref: rehabilitation hospitals)** | | |  |  |
| Nursing homes | -0.86^**^ | -162.45(-295.90, -29.01) | -0.21^**^ | -17.13(-30.66, -3.61) |
| Home and community-based care centers | 1.67^**^ | 315.68(47.91,583.45) | 1.10^**^ | 89.84(53.12,126.57) |
| **Caregivers (ref: basically trained caregivers)** | | |  |  |
| Moderately trained caregivers | -0.07 | -13.18(-47.60,21.23) | 0.06 | 4.98(-5.98,15.94) |
| Sufficiently trained caregivers | 0.36^**^ | 68.08(4.81,131.35) | 0.16^*^ | 13.20(1.39,25.02) |
| **Reimbursement rate (ref: 50%)** | |  |  |  |
| 70% | 0.02 | 3.76(-22.33,29.86) | 0.12^*^ | 10.18(-0.04,20.40) |
| 90% | 0.38^**^ | 72.66(4.29,141.03) | 0.34^**^ | 28.10(10.78,45.42) |
| **Annual premium per person (CNY)** | -0.01^*^ | — | -0.01^**^ | — |
| **Log-likelihood** | -864.23 | | -873.41 | |
| **Participants** | 201.00 | | 189.00 | |
| **Observations** | 3618.00 | | 3402.00 | |

*β, the average preferences of the study population; WTP, willingness to pay; Ref, reference level; *p＜0.05; **p＜0.01.*

| **SUPPLEMENTARY TABLE 3⎪** Subgroup analysis by attitudes to risk of dysfunction. | | | |
| --- | --- | --- | --- |
| **Attributes** | **Optimistic** | **Neutral** | **Pessimistic** |
|  | **β/WTP** | **β/WTP** | **β/WTP** |
| **Care content (ref: daily life care)** |  |  |  |
| Daily life care, rehabilitation | 0.15^*^/55.52 | -0.18/-13.35 | 0.13/5.23 |
| Daily life care, rehabilitation and emotional support | 0.22^**^/79.80 | 0.63^**^/46.64 | 0.92^**^/38.39 |
| **Care facilities (ref: rehabilitation hospitals)** | |  |  |
| Nursing homes | -0.54^**^/-196.05 | -0.60^**^/-44.09 | -0.27/-11.26 |
| Home and community-based care centers | 1.43^**^/515.07 | 1.75^**^/128.66 | 0.92^**^/38.32 |
| **Caregivers (ref: basically trained caregivers)** |  |  |  |
| Moderately trained caregivers | -0.11/-39.56 | 0.06/4.30 | 0.18/7.44 |
| Sufficiently trained caregivers | 0.18^*^/65.98 | 0.28^*^/20.88 | 0.53^**^/22.06 |
| **Reimbursement rate (ref: 50%)** |  |  |  |
| 70% | 0.08/29.30 | 0.08/5.99 | 0.13/5.25 |
| 90% | 0.29^**^/103.92 | 0.38^**^/28.13 | 0.70^**^/29.36 |
| **Annual premium per person (CNY)** | -0.003 | -0.010** | -0.020** |
| **Log likelihood** | -846.31 | -518.96 | -358.86 |
| **Participants** | 186.00 | 122.00 | 82.00 |
| **Observations** | 3348.00 | 2196.00 | 1476.00 |
| *β, the average preferences of the study population; WTP, willingness to pay; Ref, reference level; *p＜0.05; **p＜0.01.* | | | |

# Supplementary Data (two rounds of expert consultations)

**The first part: the design of attributes and levels of residents participating in long-term care insurance preference and willingness to pay research**

**(The first round)**

**Expert scoring method description:** Please judge the importance and feasibility of each item, and fill in the corresponding column in the column that you think is appropriate Points. If you think this item is unnecessary or inaccurate, please indicate "delete" in the "modification comment" column or submit a modification comment. If you think we missed something, please add it to the "recommended addition" section. Please evaluate the updated or added attribute or level's importance and feasibility. Do not leave blank or omit. (The attribute or level importance score is 1-5 points, with 5 representing the most essential and 1 the least. The feasibility points are 1-5 in order, and 5 points signify the attribute or degree. 1 point is the most difficult to operate).

1. Please rate the importance and feasibility of the following attributes in the choices box.

| **Code** | **Attribute** | **Revise opinion** | **Attribute importance** | | | | | **Attribute feasibility** | | | | |
| --- | --- | --- | --- | --- | --- | --- | --- | --- | --- | --- | --- | --- |
|  |  |  | **5** | **4** | **3** | **2** | **1** | **5** | **4** | **3** | **2** | **1** |
| 1 | Annual OOP |  |  |  |  |  |  |  |  |  |  |  |
| 2 | Security level |  |  |  |  |  |  |  |  |  |  |  |
| 3 | Service pattern |  |  |  |  |  |  |  |  |  |  |  |
| 4 | Service content |  |  |  |  |  |  |  |  |  |  |  |
| 5 | Service provider |  |  |  |  |  |  |  |  |  |  |  |
| 6 | Payment range |  |  |  |  |  |  |  |  |  |  |  |
| 7 | Service quality |  |  |  |  |  |  |  |  |  |  |  |
| Suggestions  for addition |  | | | | | | | | | | | |
|  |  | | | | | | | | | | | |
|  |  | | | | | | | | | | | |

2. Please score the importance and feasibility of the following level divisions in the choices box.

| **Attribute** | **Level** | **Revise opinion** | **Level**  **importance** | | | | | **Horizontal**  **feasibility** | | | | |
| --- | --- | --- | --- | --- | --- | --- | --- | --- | --- | --- | --- | --- |
|  |  |  | 5 | 4 | 3 | 2 | 1 | 5 | 4 | 3 | 2 | 1 |
| Annual OOP | 30 yuan |  |  |  |  |  |  |  |  |  |  |  |
|  | 36 yuan |  |  |  |  |  |  |  |  |  |  |  |
|  | 50 yuan |  |  |  |  |  |  |  |  |  |  |  |
| Security level | 70% |  |  |  |  |  |  |  |  |  |  |  |
|  | 80% |  |  |  |  |  |  |  |  |  |  |  |
|  | 90% |  |  |  |  |  |  |  |  |  |  |  |
| Service pattern | Home care |  |  |  |  |  |  |  |  |  |  |  |
|  | Institutional care |  |  |  |  |  |  |  |  |  |  |  |
|  | Community care |  |  |  |  |  |  |  |  |  |  |  |
| Service provider | Informal caregiver |  |  |  |  |  |  |  |  |  |  |  |
|  | formal caregiver |  |  |  |  |  |  |  |  |  |  |  |
| Service content | Daily life care |  |  |  |  |  |  |  |  |  |  |  |
|  | Daily life care and rehabilitation care |  |  |  |  |  |  |  |  |  |  |  |
|  | Daily life care, rehabilitation care and  psychological or spiritual comfort care |  |  |  |  |  |  |  |  |  |  |  |
| Payment range | Wide coverage |  |  |  |  |  |  |  |  |  |  |  |
|  | narrow coverage |  |  |  |  |  |  |  |  |  |  |  |
| Service quality | Provide adequate services to maintain  current health (sufficient) |  |  |  |  |  |  |  |  |  |  |  |
|  | Provide satisfactory services to  Improve health (satisfactory) |  |  |  |  |  |  |  |  |  |  |  |
| Suggestions  for addition |  | | | | | | | | | | | |
|  |  | | | | | | | | | | | |
|  |  | | | | | | | | | | | |

**The Second part: Expert Survey Form**

**Instructions for experts to fill in the form:**

In the “Judgment basis and degree of influence”column, please evaluate the extent to which each judgement base influenced your judgement (large, medium, and small), and then fill in the corresponding serial number on the horizontal line. There can be only one choice for the degree of influence of each basis. Please be sure to fill it in completely.

| **Part One Judgment Basis of Attribute and Level** | |
| --- | --- |
| Your main basis for judging attributes and level | |
| Basis for judgment | The extent to which the basis of judgment affects expert judgment |
|  | ①Large ②Medium ③Small |
| Experience ①Large ②Medium ③Small | |
| Theoretical analysis ①Large ②Medium ③Small | |
| Domestic and foreign literature ①Large ②Medium ③Small | |
| Intuitive feeling ①Large ②Medium ③Small | |
| Your familiarity with the survey content | |
| Your understanding of long-term care insurance | |
| ① Very familiar ②Rather familiar ③Generally familiar ④Not very familiar ⑤Unfamiliar | |
| Your understanding of methods of measuring preferences | |
| ① Very familiar ②Rather familiar ③Generally familiar ④Not very familiar ⑤Unfamiliar | |
| If you have any suggestions for the subject, please fill in the blank space below | |
|  | |
| **Part Two Basic Information of Experts** | |
| Name Title Work Unit | |
| Educational Background Profession Professional years | |
| Identity Number | |
| Bank card number | |
| Account bank information (please fill in the branch and bank number) | |
| Phone number | |

Thank you again for your support and help, and I wish you happy work and good health!**SUPPLEMENTARY DATA 1 ⎪**Results of the first round of expert consultations.

| **Expert** | **Attribute Importance Score** | | | | | | | |
| --- | --- | --- | --- | --- | --- | --- | --- | --- |
|  | **Annual OOP** | **Security level** | **Service pattern** | **Service content** | **Service provider** | **Service**  **content** | **Payment**  **range** | **Quality**  **of service** |
| 1 | 5.00 | 5.00 | 5.00 | 4.00 | 5.00 | 4.00 | 4.00 | 5.00 |
| 2 | 5.00 | 5.00 | 4.00 | 5.00 | 5.00 | 5.00 | 5.00 | 5.00 |
| 3 | 5.00 | 5.00 | 5.00 | 5.00 | 4.00 | 5.00 | 4.00 | 5.00 |
| 4 | 5.00 | 5.00 | 4.00 | 3.00 | 5.00 | 5.00 | 5.00 | 5.00 |
| 5 | 3.00 | 5.00 | 4.00 | 4.00 | 5.00 | 3.00 | 2.00 | 3.00 |
| 6 | 5.00 | 5.00 | 5.00 | 5.00 | 5.00 | 3.00 | 5.00 | 5.00 |
| 7 | 5.00 | 4.00 | 5.00 | 5.00 | 5.00 | 5.00 | 2.00 | 5.00 |
| 8 | 5.00 | 5.00 | 4.00 | 3.00 | 4.00 | 5.00 | 5.00 | 5.00 |
| Mean | 4.75 | 4.88 | 4.50 | 4.25 | 4.75 | 4.38 | 4.00 | 4.75 |
| Median | 5.00 | 5.00 | 4.50 | 4.50 | 5.00 | 5.00 | 4.50 | 5.00 |
| Standard deviation | 0.71 | 0.35 | 0.53 | 0.89 | 0.46 | 0.92 | 1.31 | 0.71 |
| Coefficient of variation | 0.15 | 0.07 | 0.12 | 0.21 | 0.10 | 0.21 | 0.33 | 0.15 |

**SUPPLEMENTARY DATA 2 ⎪** Experts' opinions on attributes and levels design.

| **Expert** | **Attribute** | **Attribute level** |
| --- | --- | --- |
| 1 | No modification suggestion. | No modification suggestion. |
| 2 | 1. **Security level:** From the specific connotation, it should refer to the insurance payment level. 2. **Service pattern:** It is recommended to change the service pattern to a service approach. 3. **Service provider:** Long-term care insurance services should be provided by certified professionals. 4. **Quality of service:** It is recommended to change to expected health outcomes. | 1. **Annual OOP:** It is suggested to increase the cost range appropriately, and the gap between costs is not large, which may easily lead to the problem of excessive WTP. 2. **Service pattern:** Home and community are often hard to separate. 3. **Service provider:** Long-term care insurance services should be provided by professionals, and a lot of elderly care personnel are reemployment retraining personnel, without too much medical care experience. It is recommended to distinguish between "years of practice". 4. **Quality of service:** It is suggested to modify it to maintain the existing function and improve the health status. |
| 3 | **Service content:** The scope of payment largely determines the content of the service. | 1. **Annual OOP:** There is not much difference between 30 yuan and 36 yuan. 2. **Payment range:** The level definition is vague, and the level of payment scope and the level of service content can be integrated. 3. **Quality of service:** Difficulty in operation |
| 4 | No modification suggestion. | No modification suggestion. |
| 5 | 1. **Security level:** It is more appropriate to define the attribute as the guaranteed rate. 2. **Service pattern:** Introduce the definition of each service model clearly, especially the difference between home care and community care. 3. **Quality of service:** Hard to quantify. | 1. **Annual OOP:** The horizontal gradient interval is too low, which may cause respondents to ignore the trade-off of this attribute, and the gap can be appropriately widened based on reality. 2. **Security level:** The horizontal gradient interval may still be low. 3. **Service pattern:** Institutions can be further divided into elderly care institutions and medical care institutions. In addition, home care and community care are easy to confuse, so the definition of community care should be added to the definition. 4. **Service content:** Simply display. 5. **Quality of service:** The description of the level is not easy to understand, it is recommended to change to a more straightforward one. |
| 6 | No modification suggestion. | 1. **Annual OOP:** The gap between these three levels is too small, and it is best to provide a basis for setting these three levels. 2. **Security level:** Whether the level of the above-mentioned out-of-pocket premium can reach this level of protection. 3. **Payment range:** What is the difference between payment scope and service content? Different people may have very different understandings of width and narrowness. 4. **Quality of service:** The quality of service options is difficult to quantify, and there is a certain overlap with the previous service providers and scope of services. |
| 7 | No modification suggestion. | 1. **Annual OOP:** It is recommended to readjust insurance levels by calculating WTP for a small population after the Pilot study. 2. **Security level:** Premium levels can be combined with premiums because they are aligned. 3. **Payment range:** You can define it in the preface, for example, ＂wide＂ refers to what is included and what is＂narrow＂. 4. **Quality of service:** Adequate and satisfactory are indistinguishable and undefined. The quality of service can be modified to another level. |
| 8 | 1. **Annual OOP:** The financing structure of insurance should be considered. 2. **Service Provider:** Authorities such as governments or insurance institutions may influence willingness to pay. | 1. **OOP/year:** The majority of respondents aged 45 and older were not discriminated against; the Too low amount of funding will reduce the willingness of participants. 2. **Security level:** The setting of guarantee level shall refer to countries such as Japan that have long implemented LTC and shall be determined according to their guarantee level. 3. **Payment range:** Refer to existing insurance coverage. |

**Attributes and Level Design of Research on Residents’ Long-term Care Insurance Choice Preference and Willingness to Pay**

**Expert Consultation Form (The Second round)**

Dear experts: Hello!

Thank you very much for participating in the questionnaire consultation in your busy schedule. Sincerely thank you for your enthusiastic help and guidance in this research in the first round of expert consultation! Based on the valuable opinions put forward by the experts, we adjusted and modified the corresponding attributes and levels, and formed the second round of expert consultation questionnaires. The purpose of this expert consultation is to further determine the rationality of the attributes and levels of long-term care insurance. In this round, please score the importance and feasibility of each attribute again, and make a judgment on the rationality of the attribute level setting. Your valuable opinions are very important for the scientificity of the questionnaire formulation. Thank you again for your guidance and help in our research!

Due to the needs of the subject research, please feed us your comments again within one week after receiving the inquiry form. If you have any questions about the issues covered in the inquiry form, you are very welcome, and thank you for contacting us. We are very grateful for your great support!

Research Group on Long-term Care Insurance Preference and Willingness to Pay

October 22, 2020

**SUPPLEMENTARY DATA 3⎪** Revised results of the first round of expert consultation.

| **Attribute** | **Level** | **Level importance** | | **Horizontal feasibility** | | **Expert opinion** |
| --- | --- | --- | --- | --- | --- | --- |
|  |  | $\mathbf{X}\mathbf{S}$ | **CV** | $\mathbf{X}\mathbf{S}$ | **CV** |  |
| Annual OOP | 30 yuan | 4.75±0.71 | 0.15 | 4.88±0.35 | 0.08 | The amount of funding was low, and the level gap was small. It was recommended that the gap be appropriately widened on a realistic basis; provide a basis for level setting. |
|  | 36 yuan |  |  |  |  |  |
|  | 50 yuan |  |  |  |  |  |
| Security Level | 70% | 4.88±0.35 | 0.07 | 5.00±0 | 0 | It was recommended to define the attribute as the insurance coverage rate; the horizontal gradient interval was small. |
|  | 80% |  |  |  |  |  |
|  | 90% |  |  |  |  |  |
| Service Pattern | Home care | 4.50±0.53 | 0.12 | 4.63±0.52 | 0.11 | It was recommended to further divide institutions into elderly care institutions and medical institutions; “model” had a broader meaning, and it was recommended to change to “service mode”; in reality, it was difficult to distinguish between home care and community care. |
|  | Institutional care |  |  |  |  |  |
|  | Community care |  |  |  |  |  |
| Service Provider | Informal caregiver | 4.25±0.89 | 0.21 | 4.25±0.71 | 0.17 | The establishment of long-term care insurance should be provided by trained professionals. Most elderly care workers are employed in training and have little medical care experience. It was recommended to distinguish them by "employment years". |
|  | Formal caregiver |  |  |  |  |  |
| Service Content | Daily life care | 4.75±0.46 | 0.10 | 4.38±0.92 | 0.21 | Simplify level expression |
|  | Rehabilitation care |  |  |  |  |  |
|  | Daily life care, rehabilitation care and psychological or spiritual comfort care |  |  |  |  |  |
| Payment Range | Wide coverage | 4.38±0.92 | 0.21 | 3.75±0.71 | 0.19 | The width is difficult to define, and the actual operability should not be high; it was recommended to integrate it with the service content level. |
|  | Narrow coverage |  |  |  |  |  |
| Service Quality | Provide adequate services to  maintain current health (sufficient) | 4.00±1.31 | 0.33 | 2.875±1.25 | 0.43 | It was difficult to quantify and operate actually. |
|  | Provide satisfactory services to  improve health (satisfactory) |  |  |  |  |  |

**The Second part: Expert consultation on the attributes and level design of "Residents' Long-term Care Insurance Choice Preference and Willingness to Pay"**

**(The** **second round)**

**Expert scoring method description:**

Please judge the importance and feasibility of each attribute, and fill in the corresponding score in the column that you think is appropriate. If you think the attribute is unnecessary or inaccurate, please indicate "delete" in the "modification comment" column or submit a modification comment. If you think we still have something that we haven't considered, please add it to the "recommended addition" column. Please judge the importance and feasibility of the modified or added attribute or level as well. Please do not leave blank or omissions. (The attribute importance score is 1-5 points in order, 5 points represent the most important attribute, and 1 point is the least important. The feasibility points are 1-5 points in order, 5 points represent the attribute that is the most operable, and 1 point is the most difficult to operate).

1. We have synthesized the opinions of experts and revised the first round of attributes as follows. Please judge the importance and feasibility of the following attributes and rate them in the options box.

| **Attribute** | **Revise opinion** | **Attribute importance** | | | | | **Attribute feasibility** | | | | |
| --- | --- | --- | --- | --- | --- | --- | --- | --- | --- | --- | --- |
|  |  | **5** | **4** | **3** | **2** | **1** | **5** | **4** | **3** | **2** | **1** |
| Annual premium per person |  |  |  |  |  |  |  |  |  |  |  |
| Care content |  |  |  |  |  |  |  |  |  |  |  |
| Care facilities |  |  |  |  |  |  |  |  |  |  |  |
| Caregivers |  |  |  |  |  |  |  |  |  |  |  |
| Reimbursement rate |  |  |  |  |  |  |  |  |  |  |  |
| Suggestions  for addition |  | | | | | | | | | | |
|  |  | | | | | | | | | | |
|  |  | | | | | | | | | | |

2. We have combined the opinions of experts to make the following revisions to the first round of levels. Please judge the rationality of the level settings and propose amendments.

| **Attribute** | **Level** | **Is the level appropriate?** | **Proposal for level modification** |
| --- | --- | --- | --- |
| Annual premium per person | 10 yuan |  |  |
|  | 21 yuan |  |  |
|  | 35 yuan |  |  |
|  | 55 yuan |  |  |
|  | 106 yuan |  |  |
| Care content | Daily life care |  |  |
|  | Rehabilitation |  |  |
|  | Daily life care and rehabilitation |  |  |
| Care facilities | Rehabilitation hospitals |  |  |
|  | Nursing homes |  |  |
|  | Home and community-based care centers |  |  |
| Caregivers | Basically trained caregivers |  |  |
|  | Moderately trained caregivers |  |  |
|  | Sufficiently trained caregivers |  |  |
| Reimbursement rate | 50% |  |  |
|  | 70% |  |  |
|  | 90% |  |  |
| Suggestions for addition |  | | |
|  |  | | |

3. In the "Attribute and Level Judgment Basis and Degree of Influence" column, please make a self-evaluation on the degree of influence of each judgment which your judgment criterion had (large, medium, small) and familiarity with the survey content (very familiar, relatively familiar, generally familiar, unfamiliar, unfamiliar), then fill in the corresponding serial number on the horizontal line, please be sure to fill it in completely.

| **Expert name：** | |
| --- | --- |
| **Judgment basis for attributes and levels** | |
| **1. The main basis for your judgment of attributes and level:** | |
| Basis for judgment | The extent to which the basis of judgment affects expert judgment |
|  | ① Large ②Medium ③Small |
| Practical experience ① Large ②Medium ③Small | |
| theoretical analysis ① Large ②Medium ③Small | |
| Domestic and foreign literature reference ① Large ②Medium ③Small | |
| Intuitive feeling ① Large ②Medium ③Small | |
| **2. Your familiarity with the survey content:** | |
| **2.1 Your familiarity with long-term care insurance** | |
| ① Very familiar ②Rather familiar ③Generally familiar ④Not very familiar ⑤Unfamiliar | |
| **2.2 Your understanding of methods of measuring preferences** | |
| **3. If you have any suggestions for the subject, please fill in the blank space below** | |
| ① Very familiar ②Rather familiar ③Generally familiar ④Not very familiar ⑤unfamiliar | |
|  | |

Thank you again for your support and help, and I wish you happy work and good health!

**SUPPLEMENTARY DATA 4 ⎪** Experts' opinions on attribute and level design.

| **Expert** | **Attribute** | **Attribute level** |
| --- | --- | --- |
| 1 | No modification suggestion. | **Care content:** Psychological service content was recommended to add. |
| 2 | Criteria for Long-term Care Objects  were suggested to add. | **Care facilities:** Community-home care model was suggested to consider. |
| 3 | No modification suggestion. | **Care facilities:** It seems that institutions combining medical care and nursing care cannot be completely distinguished from institutions for the elderly and medical treatment. |
| 4 | No modification suggestion. | **1. Care content:** It was suggested to readjust the level of care content on the bases of literature reading.  **2. Annual premium per person:** It was recommended that the level of the attribute be adjusted to a multiple of 10 and further adjusted to meet the linearity criterion according to the pilot study.  **3. Reimbursement rate:** The number of levels was suggested to increase and modify according to the pilot study. |
| 5 | No modification suggestion. | **1. Care facilities:** It was suggested to set the level as home care, nursing homes, geriatric hospitals/home care, and integrated medical and nursing institutions.  **2. Annual premium per person:** It was suggested to change it to "$30, $60, $90".  **3. Reimbursement rate:** It was suggested to change it to "60%, 75%, 90%". |
| 6 | No modification suggestion. | **Care content:** Medical rehabilitation nursing covered by medical insurance should be defined. |
| 7 | No modification suggestion. | **1. Annual premium per person:** It was suggested that the low-level gap should be widened and the high-level gap should be narrowed.  **2. Reimbursement rate:** A 30% increase was recommended for the corresponding care facilities. |
